# Beyond the clinic: the rise of wearables and smartphones in decentralising healthcare

**DOI:** 10.1038/s41746-023-00971-z

**Published:** 2023-11-25

**Authors:** Conor Wall, Victoria Hetherington, Alan Godfrey

**Affiliations:** 1 Department of Computer and Information Sciences, Northumbria University, Newcastle upon Tyne, UK; 2 Cumbria, Northumberland Tyne and Wear NHS Foundation Trust, Wolfson Research Centre, Campus for Ageing and Vitality, Newcastle upon Tyne, UK

**Keywords:** Computer science

## Abstract

Navigating contemporary healthcare, wearable technology and smartphones are marking the dawn of a transformative era in patient observation and personalised care. Wearables, equipped with various sensing technologies (e.g., accelerometer for movement, optics for heart rate), are increasingly being recognised for their expansive potential in (remote) patient monitoring, diagnostics, and therapeutic applications which suggests a plausible move towards a more decentralised healthcare system. This shift is evident as healthcare providers and patients alike are becoming increasingly accepting of wearable-driven tools, as they enable continuous health monitoring outside of traditional clinical settings. Equally, the ubiquitous nature of smartphones, now more than mere communication tools, is being harnessed to serve as pivotal health monitoring instruments. Their added sensing capabilities with Internet of Things (IoT) driven connectivity enable a (relatively) seamless transition from conventional health practices to a more interconnected, digital age. However, this evolving landscape is not without its challenges, with concerns surrounding data privacy, security, and ensuring equitable access to digital advances. As we delve deeper into digital healthcare, we must harness the full potential of those technologies and ensure their ethical and equitable implementation, envisioning a future where healthcare is not just hospital-centric but is part of our daily lives.

## Introduction: The emerging realistic role of wearables

In the modern healthcare landscape, wearable technology is pioneering a significant yet practical shift in patient observation and care. Recently, a *Perspective* published in this journal delved deep into the potential of wearables, highlighting their growing presence for monitoring, diagnostic, and therapeutic use, paving the way for a decentralised approach to healthcare^1^. The referenced article underscores the importance of wearables designed and tailored to meet distinct health conditions, seamlessly integrating with existing health data ecosystems while offering a cost-effective yet highly efficient alternative to monitoring a patient’s condition. The crux of the referenced *Perspective* is how the integration of wearables within healthcare could offer more personalised and timely features such as individualised health guidance, appointment notification, targeted digital health education, and assisted automated medication dosing. Interestingly, the *Perspective* demonstrates examples of health institutions such as Kaiser Permanente and Ochsner that stand out with their cutting-edge at-home digital health monitoring for blood glucose and blood pressure, the latter synchronised with electronic health records to facilitate effective management of diabetes and hypertension. Also highlighted in the perspective are smartphones, which are becoming increasingly indispensable. Smartphones, ubiquitous in their coverage, act as (near ideal) conduits between wearables and healthcare providers. The integration of smartphones could facilitate real-time data collection, decentralised analysis, and immediate feedback, e.g., empowering patients with instantaneous insights and/or remote monitoring capabilities for the medic to enter the home/community.

## Smartphones: a new frontier in health monitoring

Though smartphones are described as showing utility when integrated with wearables, their potential is further magnified when we consider them as standalone health monitoring devices^2^. They themselves are equipped with contemporary sensors and Internet of Things (IoT) enabled technology, holding the potential to bridge the gap between traditional health monitoring and (more targeted digital) medicine. The widespread presence of smartphones in our daily lives (perhaps, more than wearables) means they could offer cost-saving implications for patients and healthcare providers. However, it must be noted there are trade-offs between how physiological data is quantified between smartphones and wearables. Specifically, there may be no difference in the accuracy of data arising from both devices at a sample level (i.e., raw data). Yet, for example, if a smartphone is placed on a table while in contrast a wrist-based wearable is constantly worn, then quantified walking will differ. What must be appreciated is that one size doesn’t fit all, and to ensure continuous data collection to better inform practices in digital medicine, flexibility and device interoperability is key.

Smartphones could also enable a more interconnected and responsive health ecosystem^3^. By harnessing processing power, IoT functionality, and embedded sensor data, smartphones could function akin to advanced wearable systems: capturing, analysing, and providing real-time health metrics, thus delivering comprehensive insights and seamless feedback^4^. Consider that many carry a smartphone in their pocket and a future where it could detect subtle physical alterations, potentially even before a trained clinician observes any discernible signs. Those early detections could enable prompt medical attention or decrease the dangers of unexpected health events and avert potential fatalities. Furthermore, synchronising smartphones with online medical services could establish direct connections between patients and medical experts. Consider the hypothetical pervasive/ubiquitous digital medicine approach of smartphones as tools to detect any potential negative health outcome(s). A healthcare professional could be alerted in real-time, facilitating a digital check-up *on the fly*. Such instantaneous healthcare access could enable/facilitate a patient’s immediate need(s) but also reduce pressure on the larger healthcare system. The inherent cost-effectiveness of that hypothetical model, due to the ubiquitous nature of smartphones, implies that many challenges could be addressed without burdensome clinical trips, helping patients and hospitals to be frugal^5–7^. Before one could possibly suggest how frugality may be realised from the hypothesis, there are elephants in the room that warrant discussion.

## Decentralised challenges with opportunities

Typically, the use of any decentralised approach, especially with the patient’s own technology, raises privacy and data security concerns. Although both are well documented, it is always worthwhile noting that big (health) data generated by any digital technology are incredibly valuable for medical research but there is the potential for misuse. It is imperative that as we dive deeper into digital medicine, robust technical standards are established and maintained. Furthermore, transparency in how health data is collected and stored is vital. While administrators require a certain degree of control for oversight, it is equally important to empower users by allowing them to have clear control over their own health data^8^. For example, it could help and empower communities of patient groups to share common challenges through lived experiences or gamified approaches to rehabilitation.

There are also challenges to digital health literacy and accessibility in decentralised approaches with many digital technologies. It is essential to ensure that all users, regardless of, e.g., age, socio-economic status, or educational background, can navigate and benefit from digital tools. That may mean the provision of adequate training and user-friendly interfaces while ensuring the cost of technology does not become a barrier. Equitable access to digital resources is vital to ensure telerehabilitation reaches every individual in need^9^. Perhaps, the use of the patient’s own familiar technology (in this case, a smartphone) could overcome challenges raised here. That can be achieved through smartphone-based apps which have demonstratable usefulness to help monitor and evaluate people with diabetes and hypertension^10,11^, but those studies remain the tip of the smartphone capability iceberg. Why? Smartphones are like a multi-tool and apps the mechanism to leverage many of the onboard sensing capabilities. For example, smartphones have been highlighted to provide holistic sensing, e.g., voice, posture, gait, finger tapping, and response time in Parkinson’s disease (PD)^12^. Using that suite of sensing could have pragmatic implications for PD management. How?

## Smartphone-based rehabilitation

Smartphone-based treatment algorithms using movement sensors (i.e., accelerometers) are envisaged as preferable to generate suitable interventions within PD^13^. Why? PD can induce irregular gait/walking patterns which lead to a higher risk of falling, increased mortality and reduced quality of life^14^. Studies have explored the ability of smartphones to examine gait in people with PD^15^. By harnessing embedded movement sensors in smartphones, healthcare providers could monitor and analyse gait patterns in the home in (near) real time. That could enable the detection of (subtle) gait deficits, which could track PD progression or response to an intervention^16^. For example, personalised gait cueing mechanisms could be implemented within gait rehabilitation strategies to reduce fall risk. Specifically, personalised auditory cues could be delivered through the smartphone to assist timing and rhythm of steps. That could facilitate community-based rehabilitation with a familiar (person’s own smartphone) and routine technology from the comfort of the home^17^. That suggests a pragmatic approach to decentralised healthcare in an equitable manner.

Interestingly, auditory cueing can involve many techniques for improved delivery. Biofeedback and sonification are possible avenues of investigation. For example, sonification has been shown to influence gait characteristics^18–20^ with use as an intervention to significantly reduce freezing-of-gait, and improve motor balance in PD^11,12^, Yet, biofeedback and sonification may be particularly beneficial as a mechanism of personalisation to increase patient comfort and adherence. The latter is a notable challenge for those where a scenario of no supervised rehabilitation is commonplace, i.e., increased isolation can mean reduced motivation^13^. The use of smartphone-based cueing with biofeedback and sonification with gamification through personalised goals and/or social connectivity could effectively perform as a contemporary approach for gait (tele) rehabilitation. Specifically, it is the gamification process that is key to ensuring people stay engaged and motivated.

## Concluding thoughts

The evolution of smartphones from mere communication devices to potent tools for health monitoring and telemedicine could provide a ground-breaking shift in healthcare delivery. These capabilities are not just theoretical but have been demonstrated in practical applications (e.g., monitoring and rehabilitation of gait in people with PD). The example discussed herein showcases the power of smartphones in truly democratising at-home/habitual healthcare. As we navigate the digital medicine landscape, the importance of safeguarding sensitive health data cannot be overstated. Simultaneously, it is imperative to bridge the digital divide, ensuring the benefits of smartphone-based health monitoring and telerehabilitation are universally accessible. As we continue to innovate and explore the possibilities, we move closer to a scenario where healthcare is not confined to hospitals but reaches every individual, regardless of location or circumstance.
